# Altered Interactions between the Gut Microbiome and Colonic Mucosa Precede Polyposis in APC^Min/+^ Mice

**DOI:** 10.1371/journal.pone.0127985

**Published:** 2015-06-29

**Authors:** Joshua S. Son, Shanawaj Khair, Donald W. Pettet, Nengtai Ouyang, Xinyu Tian, Yuanhao Zhang, Wei Zhu, Gerardo G. Mackenzie, Charles E. Robertson, Diana Ir, Daniel N. Frank, Basil Rigas, Ellen Li

**Affiliations:** 1 Department of Medicine, Stony Brook University, Stony Brook, NY, United States of America; 2 Department of Applied Mathematics and Statistics, Stony Brook University, Stony Brook, NY, United States of America; 3 Department of Preventive Medicine, Stony Brook University, Stony Brook, NY, United States of America; 4 Department of Medicine, University of Colorado Anschutz Medical Campus, Aurora, CO, United States of America; Charité, Campus Benjamin Franklin, GERMANY

## Abstract

Mutation of the adenomatous polyposis coli (APC gene), an early event in the adenoma-carcinoma sequence, is present in 70-80% of sporadic human colorectal adenomas and carcinomas. To test the hypothesis that mutation of the APC gene alters microbial interactions with host intestinal mucosa prior to the development of polyposis, culture-independent methods (targeted qPCR assays and Illumina sequencing of the 16S rRNA gene V1V2 hypervariable region) were used to compare the intestinal microbial composition of 30 six-week old C57BL/6 APC^Min/+^ and 30 congenic wild type (WT) mice. The results demonstrate that similar to 12-14 week old APC^Min/+^ mice with intestinal neoplasia, 6 week old APC^Min/+^ mice with no detectable neoplasia, exhibit an increased relative abundance of *Bacteroidetes* spp in the colon. Parallel mouse RNA sequence analysis, conducted on a subset of proximal colonic RNA samples (6 APC^Min/+^, 6 WT) revealed 130 differentially expressed genes (DEGs, fold change ≥ 2, FDR <0.05). Hierarchical clustering of the DEGs was carried out by using 1-r dissimilarity measurement, where r stands for the Pearson correlation, and Ward minimum variance linkage, in order to reduce the number of input variables. When the cluster centroids (medians) were included along with APC genotype as input variables in a negative binomial (NB) regression model, four of seven mouse gene clusters, in addition to APC genotype, were significantly associated with the increased relative abundance of Bacteroidetes spp. Three of the four clusters include several downregulated genes encoding immunoglobulin variable regions and non-protein coding RNAs. These results support the concept that mutation of the APC gene alters colonic-microbial interactions prior to polyposis. It remains to be determined whether interventions directed at ameliorating dysbiosis in APC^Min/+^mice, such as through probiotics, prebiotics or antibiotics, could reduce tumor formation.

## Introduction

Alterations in the gut microbiome (dysbiosis) have been reported in human colonic neoplasia [1–6]. However it remains unclear as to whether dysbiosis represents a response to tumorigenesis or whether it precedes tumor formation. One of the most prominent genetic mutations associated with the pathogenesis of sporadic and hereditary colorectal cancers (CRC) lies in the tumor suppressing adenomatous polyposis coli (APC) gene [7–13]. A germ-line mutation of the APC gene causes familial adenomatous polyposis (FAP), which results in the development of multiple colorectal adenomas at an early age that unequivocally lead to CRC if no surgical interventions are taken. APC mutations also represent an early event in the adenoma-carcinoma sequence and are present in about 70–80% of sporadic human colorectal adenomas and carcinomas.

The multiple intestinal neoplasia (Min) mouse model of FAP carries a truncation mutation at codon 850 of the *Apc* gene [14]. Studies comparing the number of intestinal polyps in germ-free and conventionally raised C57Bl/6 APC^Min/+^ mice suggest that the gut microbiome may promote development of intestinal neoplasia [15, 16]. One study reported decreased incidence of polyps in only the mid small intestinal segment, however a subsequent study reported a significant reduction of intestinal adenomas in both the small and large intestine of germ-free mice compared with conventionally raised mice. Antibiotic treatment of C57BL/6 APC^Min/+^MSH2^-/-^mice, which carry both the APC mutation and an HNPCC DNA mismatch repair mutation, reduced the number of polyps in both the small and large intestine [17].

We hypothesize that mutation of the APC gene results in alterations in host-microbiota interactions prior to tumor formation. To test this hypothesis, gut microbial composition was compared between 6 week-old C57Bl/6 APC^Min/+^, prior to the development of detectable neoplasia [18], and congenic WT mice.

## Materials and Methods

### Animal Type and Housing

All of the mice were acclimated for two weeks in order to reduce stress from traveling. Carbon dioxide was used during euthanasia of the mice. This study was approved by the Institutional Animal Care and Use Committee (#202449) and Division of Laboratory Animal Resources at Stony Brook University. Three shipments of 10 four-week-old female C57BL/6J APC^Min/+^ and 10 four-week-old female C57BL/6J WT mice were received from The Jackson Laboratory (Bar Harbor, ME) between June 2012 and May 2013. APC^Min/+^ mice and WT mice were housed separately in groups of three to four in specific pathogen free (SPF) cages for two weeks prior to euthanization. All the experiments strictly followed guidelines from the Institutional Animal Care and Use Committee and Division of Laboratory Animal Resources at Stony Brook University.

### Tissue and Luminal Content Sample Collection

All of the mice were euthanized at 6 weeks of age using carbon dioxide. Immediately after sacrifice, the gastrointestinal tract was divided along its cephalocaudal axis as previously described [19]. The segments analyzed included the ileum, cecum, proximal colon, and distal colon. Each small intestinal segment was washed in sterile phosphate buffered saline to remove the luminal content. A 1.0–1.5-cm section was obtained from the proximal ends of duodenum, jejunum, distal ends of ileum, proximal colon, and distal colon, and placed into RNAlater solution (Life Technologies, Grand Island, NY, USA) for RNA/DNA studies. The cecum was placed in its entirety in RNAlater. Three pellets of distal colonic luminal content (formed stool) were collected from the distal colon and stored in RNAlater. All the samples were kept at room temperature overnight and then stored in -80°C. In the first cohort, the remainder of each intestinal segment was processed into “swiss rolls” and fixed in 10% buffered formalin for histological analyses. In the second and third cohort, the remainder of each intestinal segment was stained with 0.25% methylene blue and inspected under a dissecting microscope for adenomas and aberrant crypt foci [20]. Stools were collected from nine 12–14 week APC^Min\+^ female mice with intestinal neoplasia [18] and six WT female mice, and placed into RNAlater.

### DNA and RNA Extraction of Intestinal Tissue and Luminal Content Samples

Total RNA and DNA, (host and associated bacterial mixed community) were extracted from the duodenum, jejunum, ileum, cecal pouch, proximal colon and distal colon tissues using TRI Reagent (Sigma, St. Louis, MO) according to the manufacturer’s recommendations. For distal colonic luminal content samples, DNA was extracted using the UltraClean Fecal Kit (Mo BIO Laboratories, Inc., Carlsbad, CA).

### Quantitative PCR (qPCR) for targeted bacterial clades

QPCR assays were performed using established primers for *Bacteroides–Prevotella–Porphyromonas* [21], *Lachnospiraceae* [21], and total bacteria [22] on all tissue and distal colonic luminal content samples as previously described [23, 24]. The relative abundance of taxa within the Bacteroidetes phylum measured by ΔCt = Ct (threshold cycle)_total bacteria_ - Ct_*Bacteroides–Prevotella–Porphyromonas*_. The relative abundance of taxa within the *Lachnospiraceae* clade (i.e., Clostridia Group Xi’an clade) was measured by ΔCt = Ct (threshold cycle)_total bacteria_ - Ct_*Lachnospiraceae*_ as previously described [23, 24]. All assays were carried out in triplicate. Plasmid quantification standards were prepared from representative clones of the target organisms. The Mann-Whitney test using GraphPad Prism 5 (La Jolla, California) was performed to compare APC^Min/+^ and WT ΔCt values. The Bonferroni correction was made to correct for multiple comparison, thus significance required p< 0.025.

### 16S rRNA Amplicon Library Construction and Illumina V1V2 Sequencing Analysis

Bacterial profiles were determined by broad-range amplification and sequence analysis of 16S rRNA genes following our previously described methods [25, 26]. In brief, amplicons were generated using primers that target approximately 300 bp. of the V1V2 variable region of the 16S rRNA gene (primers 8F and 338R, modified by addition of Illumina adapter and dual index sequences). PCR products were normalized using a SequalPrep kit (Invitrogen, Carlsbad, CA), pooled, lyophilized, purified and concentrated using a DNA Clean and Concentrator Kit (Zymo, Irvine, CA). Pooled amplicons was quantified using Qubit Fluorometer 2.0 (Invitrogen, Carlsbad, CA). The pool was diluted to 4nM and denatured with 0.2 N NaOH at room temperature. The denatured DNA was diluted to 15pM and spiked with 25% of the Illumina PhiX control DNA prior to loading the sequencer. Illumina paired-end sequencing was performed on the Miseq platform with version v2.3.0.8 of the Miseq Control Software and version v2.3.32 of MiSeq Reporter, using a 600 cycle version 3 reagent kit.

Paired-end sequences were sorted by sample via barcodes in the paired reads with a python script [25]. Sorted paired end sequence data were deposited in the NCBI Short Read Archive under BioProject Accession Number: PRJNA270112 (www.ncbi.nlm.nih.gov/bioproject/PRJNA270112)PRJNA270112. The sorted paired reads were assembled using phrap [27, 28] and paired reads that did not assemble were discarded. Assembled sequence ends were trimmed over a moving window of 5 nucleotides until average quality met or exceeded 20. Trimmed contigs with more than 1 ambiguity or shorter than 200 nt were discarded. Potential chimeras identified with Uchime (usearch6.0.203_i86linux32) [29] using the Schloss [30] Silva reference sequences were removed from subsequent analyses. Assembled sequences were aligned and classified with SINA (1.2.11) using the 418,497 bacterial sequences in Silva 115NR99 as reference configured to yield the Silva taxonomy [31, 32]. Operational taxonomic units (OTUs) were produced by clustering sequences with identical taxonomic assignments. OTU counts were normalized between samples by dividing sequence counts by the total number of sequences generated per sample. Phylum-level and family-level OTU tables were generated by collapsing lower level OTUs into higher-level categories. OTUs with a relative abundance <0.0001 and with a prevalence <0.01 were culled and relative abundances then transformed using the square root function [33]. The software package Explicet (v2.9.4, www.explicet.org) was used to display OTU data and estimate alpha diversity indices (i.e., S_Chao1_, Shannon complexity [H], and Shannon Evenness [H/H_max_]) through 1000 replicate samplings of rarefied datasets [34].

Comparisons of individual phyla and families passing the initial filtering step, were conducted as follows. Because of the commonly observed over-dispersion in microbiome count data [35], the effects of APC^Min/+^ genotype on individual OTU abundances were examined using the negative binomial (NB) regression model as follows:

log(μijk)=(βi0)k+β1kgenotypeij+(log total count)ij(βi0)k=b0k+bikI{shipmentij=i}Yijk~NB(μijk,ϕk)

Y_ijk_ denotes the OTU k’s observed count for mouse j in shipment i, *μ*_*ijk*_ is the mean parameter for OTU k’s count distribution of mouse j in shipment I and *ϕ*_*k*_ is the dispersion parameter. Shipment refers to three different deliveries of mice (mice 1–20, mice 21–40 and mice 41–60), which has a zero-mean random coefficient. Coefficients b_0_ and *β*_1_ are fixed constant representing grand mean and APC genotype respectively. The log total sequence count for each mouse is considered as an offset. In addition, the skewness of the distribution of low abundance OTUs causes a large proportion of zero counts. Therefore, a zero-inflated version of the negative binomial (NB) model is also fitted to OTUs with zero counts in additional to the NB model:

log(μijk)=(βi0)k+β1kgenotypeij+(log total count)ij(βi0)k=b0k+bikI{shipmentij=i}Yijk~NB(μijk,ϕk)(Yijk|Xijk=0)=0,(Yijk|Xijk=1)=YijkXijk~ Bernoulli(π)

The NB and zero-inflated NB models are chosen based on AIC criterion [36]. The p values for the genotype effects on each OTU were then adjusted by the Benjamini-Hochberg [37] procedure to calculate the FDR. Significance was set as FDR<0.05. In addition, a 10-fold cross-validation was performed to validate the significant OTUs that were identified. The square root transformation was then applied to the relative abundances to correct for the skewness and to reduce the coefficient of variation. Comparisons of overall microbial composition between APC^Min/+^mice versus the wild type mice were subsequently conducted using the permutation Hotelling T2 test with 10,000 permutations using the R package ‘Hotelling’ [38]. Principle coordinate analysis (PCoA) was conducted at the lowest taxonomic level (genus) using the wcmdscale function implemented by the vegan R package [39] and using Morisita-Horn dissimilarity scores.

### Histological Analysis of the Intestinal Sections

Histological analysis was carried out in a subset of 10 APC^Min/+^ mice and 10 wild type mice (1^st^ cohort) by constructing “Swiss rolls” of intestinal segments. These segments were stained with hematoxylin and eosin and scored for adenomas and inflammation by a pathologist who was blinded with respect to the genotype of the mice (N.O.), as previously described [40]. Inspection of intestinal segments stained with 0.25%methylene blue was carried out on the second and third cohorts of mice with the aid of a Zeiss dissecting scope for detection of adenomas and aberrant crypt foci [20].

### Comparison of Mouse Proximal Colonic mRNA Expression in APCMin/+ and Wild type Mice

IL-1β mRNA expression relative to actin mRNA, was measured in cecal, proximal colonic, and distal colonic intestinal tissue RNA samples in all 30 APC^Min/+^ and 30 WT mice as previously described [8]. RNA extracted from the proximal colon of 9 week-old WT mice treated with 3% DSS in water for 7 days was used as a positive control for the assay. The IL-1β ΔCt values (ΔCt = Ct_actin_-Ct_IL1β_) were compared between APC^Min/+^ and WT groups using the Mann-Whitney test. Significance was set at a threshold of p <0.05.

Aliquots (1 μg) of proximal colon RNA samples from 6 APC were subjected to paired-ends 100 bp Illumina sequencing. The RNA-Seq libraries were prepared and sequenced at the New York Genome Center. Between 81 and 314 million reads were generated for each of the RNA samples. The RNA-Seq data were deposited in NCBI's Gene Expression Omnibus database with accession number GSE67634. The short reads were aligned to the GRCm38 genome (http://useast.ensembl.org/Mus_musculus/Info/Annotation) using STAR (Spliced Transcripts Alignment to a Reference) [41], and then converted to raw gene counts using featureCounts [42]. The edgeR package [43] was used to identify differentially expressed (FDR<0.05) genes (DEGs) between the APC^Min/+^ and wild type mice, using additional cutoff of 2-fold differential expression between groups. Hierarchical clustering based on the reads per kilobase of exon per million mapped reads (RPKM) value of the 130 DEGs was carried out by using 1-r dissimilarity measurement and Ward linkage, and the cluster number (n = 7) was chosen based on inspection of the coefficient of determination (R2) plot as previously described [44]. Second, a negative binomial (NB) regression model was fit with gene clusters as following:

log(μijk)=βi0+β1kgenotypeij+Σhαhxijh+(log total count)ij(βi0)k=b0k+bikI{shipmentij=i}Yij~NB(μij,ϕk).

x_ijh_ is gene cluster h’s expressions centroids (medians) of mouse j in shipment i. μ_ijk_ is the mean parameter for phylum k’s count distribution of mouse j in shipment i and ϕ_k_ is the dispersion parameter. “Shipment” is as defined above. b_0_ and β_1_ are fixed coefficients representing grand mean and APC genotype respectively. The log total count of each mice is considered as an offset. Best subset model selection was conducted to choose the model with lowest AIC. All models were fitted with R package: glmmADMB [45].

## Results

The relative abundance of *Bacteroidetes spp* is increased in APC^Min/+^mice colonic mucosa and luminal content prior to the development of intestinal neoplasias.

In preliminary targeted qPCR studies, we observed that the relative abundance of *Bacteroidetes spp*. in fecal DNA was higher in 12–14 week-old APC^Min/+^ female mice compared to age-matched WT female mice (ΔCt = -2.4 vs. ΔCt = -5.1, p = 0.0004). To test the hypothesis that the increase in the relative abundance of *Bacteroidetes spp*. preceded polyposis, we compared the relative abundance of this clade in the ileal, cecal, proximal colonic, distal colonic mucosa and the distal colonic luminal content in 6 week-old mice. Because gender effects have been previously reported on the number and location of polyps, we restricted our analysis to female mice [46]. Previous studies conducted on 6 week APC^Min/+^ female mice had established the absence of detectable neoplasias at that age [18]. The absence of intestinal adenomas and aberrant crypt foci was confirmed in the mice included in this study by microscopic inspection of the intestinal segments. The mean histological scores for inflammation were 0.1 and 0 (p = 0.37) for APC^Min/+^ and WT colons, respectively (n = 10 in each category). We observed a significant increase in the relative abundance of *Bacteroidetes spp*. in proximal colonic, distal colonic and distal luminal contents between the APC^Min/+^ and WT-control mice, but no significant difference in the ileal or cecal mucosal samples (Table 1). In contrast we observed no significant difference is *Lachnospiriceae spp*., a prominent group of Firmicutes, except in the luminal content of the distal colon, which exhibited significantly higher loads in WT mice (p = 0.003).

**Table 1:** QPCR comparison of the relative abundances of the *Bacteroidetes* phylum and the *Lachnospiriceae* clade within the Firmicutes phylum in 6 week old APC^Min/+^ and WT mice.

***Bacteroidetes***	**APC** ^**Min/+**^ **ΔCt Median (range)**	**Wild type ΔCt Median (range)**	**P-value**
Ileal mucosa	-2.5 (-6.4, 0.5)	-3.0 (-7.5, 0.4)	0.65
Cecal mucosa	-4.0 (-8.0, -1.0)	-4.3 (-6.2, -3.1)	0.10
**Proximal colonic mucosa**	**-3.4 (-6.9, -1.6)**	**-5.0 (-6.4, -2.2)**	**0.0008**
**Distal colonic mucosa**	**-3.1 (-7.1, -1.6)**	**-3.9 (-7.9, -1.7)**	**0.005**
**Distal colonic luminal content**	**-1.4 (-2.6, -0.1)**	**-2.3 (-5.3, -1.0)**	**<0.0001**
***Lachnospiriciae***	**APC ΔCt Median (range)**	**Wild type ΔCt Median (range)**	**P-value**
Ileal mucosa	-4.2 (-12.1, -0.9)	-3.9 (-11.6, -2.4)	0.89
Cecal mucosa	-2.9 (-8.6, -1.3)	-2.4 (-6.1, -0.7)	0.06
Proximal colonic mucosa	-2.2 (-9.2, -0.3)	-1.9 (-11.4, -0.3)	0.15
Distal colonic mucosa	-3.6 (-8.3, -1.5)	-2.8 (-7.4, -0.5)	0.06
**Distal colonic luminal content**	**-4.8 (-9.0, -3.4)**	**-3.9 (-10.4, -1.6)**	**0.003**

The qPCR assays were conducted using established primers as described in Methods. The median and range of ΔCt values (~Log_2_ relative abundance of targeted taxa) are listed for.the ileal, cecal, proximal colonic, distal colonic mucosal samples and the distal colonic luminal samples collected from 30 6 week old APC^Min/+^ and 30 wild type mice. The p-values were carried out using the Mann-Whitney U test. The Bonferroni correction was made to the p-value, so that significance was set at p<0.025.

### 16S rRNA sequence analysis of proximal colonic mucosal samples from APC^Min/+^ and WT mice

Illumina sequencing of the 16S rRNA gene V1V2 hypervariable region was carried out for the proximal colonic DNA samples. A total of 13,248,412 high-quality sequences were generated (average sequence length: 317 nt; average sample size: 220,807 sequences/sample; minimum: 9,381 sequences; maximum: 411,636 sequences, exclusive of negative controls which were near zero). The median Good’s coverage score was ≥ 99.9987% at the rarefaction point of 9,381 sequences, indicating deep sequence coverage of the intestinal microbiome.

**Fig 1:**
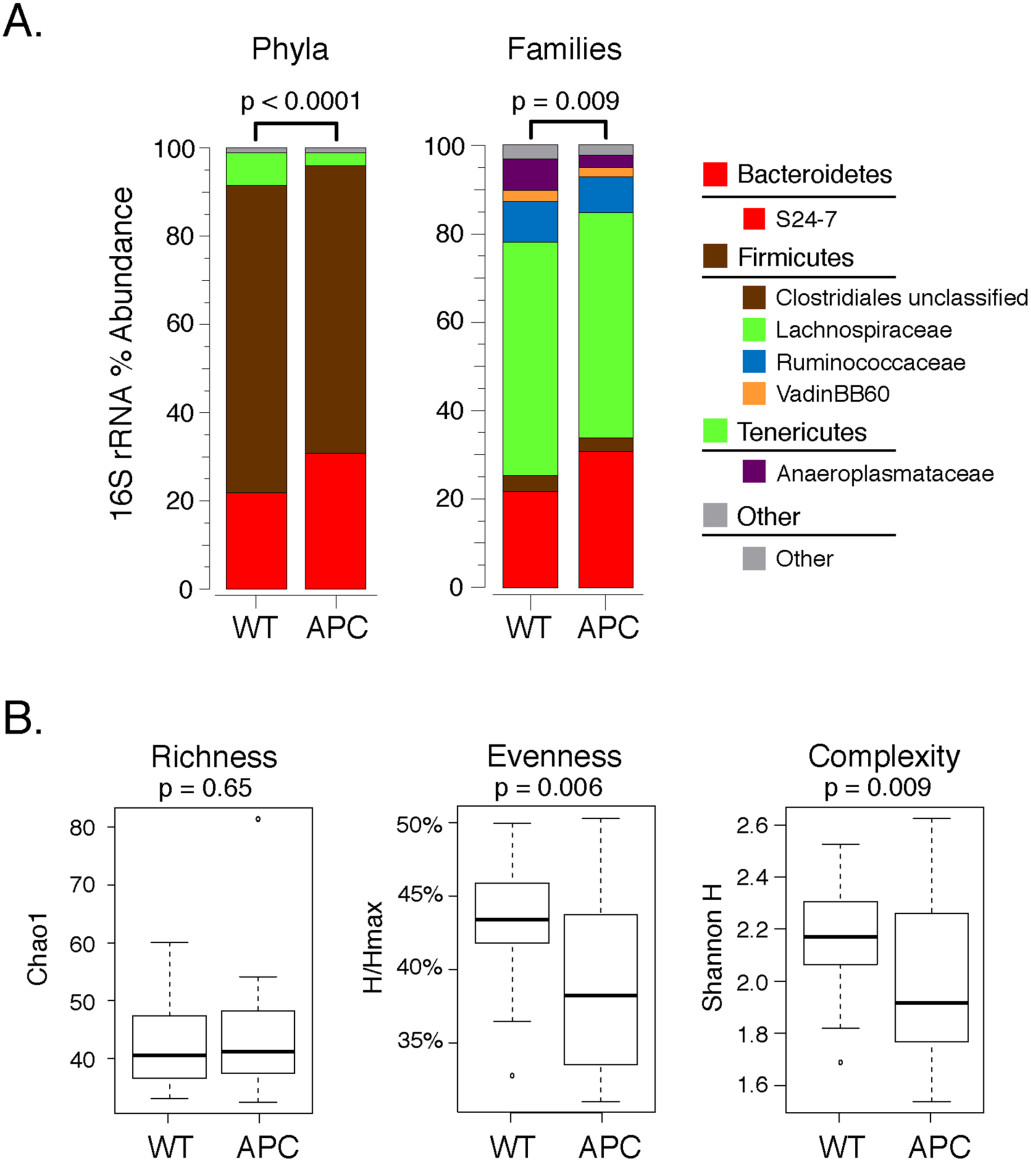
Comparison of phyla and families between wildtype (WT) and APC^Min/+^ (APC) mice. A. The mean relative abundances of phyla (left panel) and families (right panel) as inferred from the 16S rRNA sequence analysis. Only phyla and families with relative abundances >0.5% are shown. The Hotelling T2 test was used to compare the overall microbial composition, with p-values noted above each barchart.

The 16S rRNA sequencing results confirmed the targeted qPCR results in demonstrating a significant increase in the relative abundance of taxa within the Bacteroidetes phylum (FDR = 0.0009) in the APC^Min/+^ mice (Fig 1A and Table 2). Within the *Bacteroidetes* phylum, the predominant family was *S24-7*, whose relative abundance was also significantly increased in APC^Min/+^ mice (Fig 1A and Table 3, FDR = 0.0015). 16S rRNA sequencing detected a significant reduction in the relative abundance of taxa within the *Tenericutes* phylum (FDR< 0.0001), as well as a significant reduction in the relative abundance of the *Anaeroplasmataceae* family (FDR < 0.0001), which was the most prevalent family in the Tenericutes phylum. The results were also confirmed in all 10 cross-validations. The relative abundance of the *Cyanobacteria* phylum and the Chloroplast family, which is the most prevalent family in this phylum), was decreased in APC^Min/+^ mice (FDR = 0.047). However this observation was confirmed in only 4 out of 10 cross-validations.

**Table 2:** 16S rRNA Sequence comparison of the relative abundances of phyla in the proximal colonic mucosa of 6 week old APC^Min/+^ and WT mice.

Phyla	Mean Relative Abundance		Test type, P-value and FDR		
	APC	WT	Test Type	P-value	FDR
Firmicutes	0.65247	0.69756	NB regression	0.10125	0.19302
**Bacteroidetes**	0.30827	0.21810	NB regression	0.00022	0.00089
**Tenericutes**	0.029	0.07425	NB regression	0.00001	0.00008
Bacteria	0.00535	0.00672	NB regression	0.16889	0.19302
Proteobacteria	0.00432	0.00276	NB regression	0.15898	0.19302
Actinobacteria	0.00055	0.00047	NB regression	0.31061	0.31061
**Cyanobacteria**	0.00004	0.00012	NB regression	0.01753	0.04675
Verrucomicrobia	0	0.00002	NB regression	0.16798	0.19302

Seven phyla remained after preprocessing filtering (maximum relative abundance < 0.0001, prevalence <0.01). Significant differences were detected in the bolded **phyla**, with the threshold set as FDR <0.05.

**Table 3:** 16S rRNA Sequence comparison of the relative abundances of families in the proximal colonic mucosa of 6 week old APCMin/+ and WT mice.

Families	Mean Relative Abundance		Test type, P-value and FDR		
	APC	WT	Test Type	P value	FDR
Firmicutes phylum					
*Lachnospiriceae*	0.50691	0.52577	NB regression	0.4327	0.5024
*Ruminococcaceae*	0.08136	0.09148	NB regression	0.1694	0.2072
*Unassigned Clostridiales*	0.03145	0.03665	NB regression	0.0589	0.0727
*vadinBB60*	0.02152	0.02471	NB regression	0.2689	0.3203
*** Erysipelotrichaceae***	0.00369	0.00622	NB regression	0.0020	0.0027
*** Lactobacillaceae***	0.00288	0.00402	NB regression	0.0399	0.0497
*** Peptococcaceae***	0.00237	0.00650	NB regression	0.0000	0.0000
*Unassigned Firmicutes*	0.00118	0.00121	NB regression	0.9504	0.9717
*Family XIII Incerta Sedis*	0.00081	0.00076	NB regression	0.6219	0.6762
*** Peptostreptococcaceae***	0.00010	0.00012	NB regression	0.0001	0.0001
*Clostridiaceae*	0.00006	0.00007	NB regression	0.9443	0.9717
*** Paenibacillaceae***	0.00005	0.00000	NB regression	0.0000	0.0001
*Bacillaceae*	0.00004	0.00003	NB regression	0.7316	0.7831
*** Staphylococcaceae***	0.00003	0.00001	NB regression	0.0157	0.0203
*Thermoactinomycetaceae*	0.00000	0.00001	zero-inflated NB regression	0.9933	0.9975
*** Unassigned Bacilli***	0.00001	0.00000	zero-inflated NB regression	0.0000	0.0000
*Bacteroidetes phylum*					
*** S24-7***	0.30782	0.21769	NB regression	0.0002	0.0015
*** Unassigned Bacteroidales***	0.00016	0.00007	NB regression	0.0000	0.0000
*Bacteriodaceae*	0.00014	0.00018	NB regression	0.5226	0.5818
*Rickenellaceae*	0.00009	0.00008	NB regression	0.5008	0.5670
*Prevotellaceae*	0.00003	0.00003	NB regression	0.7769	0.8251
*Porphyromonadaceae*	0.00002	0.00004	NB regression	0.4825	0.5508
*Unassigned Bacteroidetes*	0.00001	0.00000	NB regression	0.6390	0.6893
Ternicutes phylum					
*** Anaeroplasmataceae***	0.02664	0.07133	NB regression	0.0000	0.0000
*RF9*	0.00236	0.00293	NB regression	0.2202	0.2647
Proteobacteria phylum					
*** Enterobacteriaceae***	0.00319	0.00039	NB regression	0.0002	0.0003
*Phyllobacteriaceae*	0.00046	0.00144	NB regression	0.9975	0.9975
*Bradyrhizobiaceae*	0.00038	0.00065	NB regression	0.8941	0.9280
*Sphingomonadaceae*	0.00008	0.00011	NB regression	0.3601	0.4253
*Methylobacteriaceae*	0.00010	0.00007	NB regression	0.8034	0.8467
*** Ricketsiella/mitochondria***	0.00001	0.00004	NB regression	0.0338	0.0425
*Burkholderiaceae*	0.00001	0.00001	NB regression	0.5053	0.5674
*Alcaligenaceae*	0.00001	0.00001	NB regression	0.5501	0.6029
*** Moraxellaceae***	0.00001	0.00001	NB regression	0.0336	0.0425
*Pseudomonadaceae*	0.00001	0.00001	NB regression	0.9914	0.9975
*** Desulfovibrionaceae***	0.00001	0.00000	NB regression	0.0120	0.0156
Actinobacteria phylum					
*Coriobacteriaceae*	0.00050	0.00042	NB regression	0.3842	0.4499
*Proprionibacteriaceae*	0.00003	0.00004	NB regression	0.4533	0.5218
*Corynebacteriaceae*	0.00000	0.00001	NB regression	0.5266	0.5818
Cyanobacteria phylum					
** Chloroplast**	0.00004	0.00012	NB regression	0.0213	0.0273
Verrucomicrobia phylum					
*Verrucomicrobiaceae*	0.00000	0.00002	zero-inflated NB regression	0.8836	0.9240
Other Phyla					
*Other Bacteria*	0.00535	0.00672	NB regression	0.2041	0.2475

Forty-two families remained after preprocessing filtering (maximum relative abundance < 0.0001, prevalence <0.01). The 42 families are listed within their respective phyla in order of their relative abundance, with the remaining families combined in Other categories. The phyla are underlined in the table. Significant differences were detected in the bolded **families**, with the threshold set as FDR <0.05.

Although 16S rRNA sequence analysis did not detect a significant difference in the overall relative abundance of the Firmicutes phylum, it also confirmed the lack of any change in the relative abundance of taxa within *Lachnospiriciae* clade, thus confirming the qPCR data (see Table 1). Furthermore decreases as well as increased were observed in some of the individual families within the Firmicutes phylum (Table 3). Similarly, although 16S rRNA sequence analysis did not detect a significant difference in the overall relative abundance of the Proteobacteria phylum, increases as well as decreases were observed in some of the individual families within the Proteobacteria phylum.

**Fig 2:**
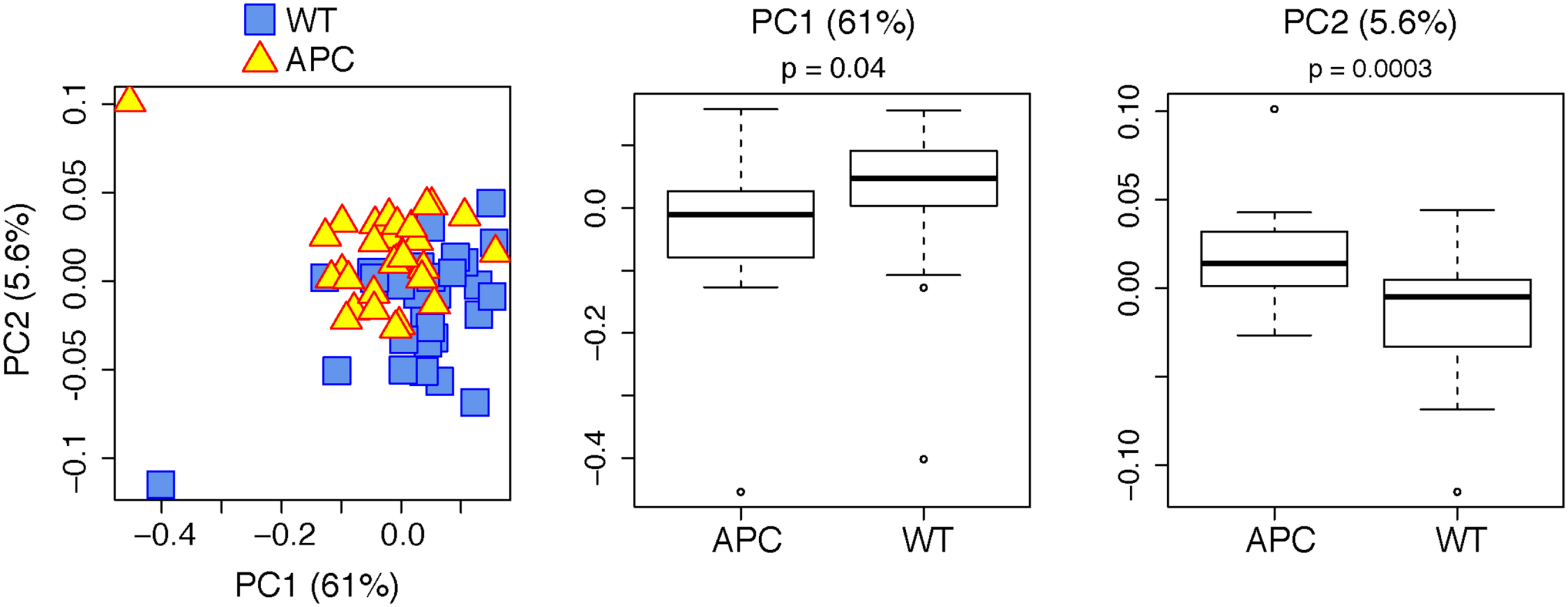
Principle Coordinate Analysis. PCoA was conducted at the family taxonomic level using a pairwise dissimilarity matrix calculated using the Morisita-Horn beta-diversity index. Each triangle is representative of a single APC^Min/+^ (APC) mouse and each square is representative of a single WT mouse, plotted along the first two principal component axes (left panel). PC1 and PC2 accounted for 61% and 5.6%, respectively, of total variance. The middle and right panels display the PC scores along axes 1 and 2, respectively. Differences in scores between genotypes were assessed by student t-test.

Overall, the proximal colonic microbiomes differed between APC^Min/+^ and WT mice at both the phylum (p<0.0001) and family (p <0.0001) levels, as assessed by a 10000-permutation Hotelling T2 test of the 7 phyla and 42 families that passed the initial filtering step (Fig 1A). The proximal colonic microbiomes of APC^Min/+^ mice were also characterized by significantly lower OTU complexity (Shannon H; p = 0.009) and evenness (Shannon H/Hmax; p = 0.006) compared with WT mice (Fig 1B); OTU richness (S_Chao1_) was comparable in the two groups. Finally, principle coordinates analysis (PCoA) demonstrated significant associations of principle component axes 1 and 2 with APC genotype (see Fig 2).

### Differentially expressed genes in 6 week-old APC^Min/+^mice

We reasoned that differences in colonic microbial composition associated with the APC^Min/+^ genotype must be linked to alterations in mouse colon gene expression, despite the lack of histological evidence of neoplasias. To examine how the APC mutation could alter the host colon gene expression, we conducted parallel RNA-sequence analysis on 6 APC^Min/+^ mice and 6 WT mice, (sampling all three cohorts). A total of 130 host genes (fold change > 2 fold, FDR <0.05) were selected using edgeR [43]. The DEGs were grouped into seven clusters as described in *Methods* (see Table 4), with 106 upregulated genes distributed among three clusters and the 24 downregulated genes distributed among four clusters.

**Table 4:** Hierarchical clustering of DEGs in APC^Min/+^ proximal colonic RNA transcripts vs. control RNA samples.

Gene name	Log_2_ FC	FDR	Gene name	Log2 FC	FDR
**Cluster 1**	**Up ↑**		**Cluster 4**	**Down↓**	
Hoxd13	7.95	5.00E-04	Rmrp	-3.9	4.00E-04
Tgm3	9.07	6.00E-04	Gm22513	-3.97	8.00E-04
Atp12a	6.4	8.00E-04	Gm26035	-5.49	8.00E-04
Ly6g	9.39	8.00E-04	Igkv5-48	-3.23	0.0022
Sval1	7.44	8.00E-04	Gm24146	-4.69	0.0023
Fut9	6.44	0.001	Igkv12-98	-5.86	0.0023
Gm8540	8.63	0.0018	Metazoa_SRP	-3.23	0.0034
AI854703	4.65	0.0022	Rn7sk	-3.04	0.0036
B3gnt7	5.27	0.0023	Igkv3-5	-5.88	0.0044
Cpn2	4.92	0.0023	Rpph1	-2.62	0.0073
Evx1	8.64	0.0023	Igkv3-1	-5.52	0.0224
Gm15053	8.81	0.0023	Vaultrc5	-2.57	0.0267
Itih2	5.16	0.0023	Ighv1-7	-2.54	0.0352
Mptx1	5.36	0.0023	Klk1b22	-2.09	0.0352
Myh2	3.28	0.0023	Olfr424	-4.87	0.0352
Thbs4	4.23	0.0023	Gm22179	-3.19	0.0353
Casp14	10.53	0.0029	Igkv9-124	-3.76	0.0445
Gm16341	5.86	0.0032	**Cluster 5**	**Down↓**	
Best4-ps	6.75	0.0034	Ighv5-15	-6.07	5.00E-04
Ctse	4.5	0.0034	Igkv2-109	-5.08	0.0066
Fxyd4	9.83	0.0034	**Cluster 6**	**Down↓**	
Gm2539	10.01	0.0034	Ighv9-4	-3.72	0.0176
Sval3	7.01	0.0034	Igkv9-120	-3.07	0.0432
Klk15	9.86	0.0035	Ighv1-80	-3.7	0.0456
Tmprss13	7.92	0.0035	**Cluster 7**	**Down↓**	
Hoxb13	10.06	0.0039	Igkv14-100	-3.87	0.0388
Hoxa13	3.92	0.0044	Sel1l2	-7.57	0.0444
Mptx2	5.49	0.0044			
Insl5	4.45	0.0045			
Pla2g4f	3.83	0.0045			
Slc28a3	4.12	0.0046			
Vsig1	3.39	0.0046			
Rims4	3.76	0.0047			
Vtcn1	5.41	0.0049			
Anxa8	3.61	0.0053			
Pdzd7	3.17	0.0053			
4930552P12Rik	3.66	0.0059			
HOXA11-AS1_5	4.62	0.0059			
Gpr83	4.27	0.006			
B3gnt5	2.98	0.0064			
Gjb5	3.34	0.0066			
Tnip3	2.94	0.0066			
Eno3	2.53	0.007			
Gjb4	3.84	0.0074			
Nxpe4	3.51	0.0074			
Spink3	3.63	0.0083			
Trpv3	3.11	0.0091			
Hoxa11os	3.58	0.0098			
2310079G19Rik	8.03	0.01			
Gm11535	7.68	0.01			
Cyp2f2	3.9	0.0112			
HOXA11-AS1_4	3.93	0.0112			
Muc1	3.11	0.0112			
St8sia5	7.08	0.0126			
HOXB13-AS1_2	7.9	0.015			
Cyp2a12	6.98	0.0156			
Ttr	2.01	0.0172			
Nccrp1	3.85	0.0173			
Evx2	7.43	0.0187			
Slc46a1	2.38	0.0187			
Csta	4.42	0.0188			
Gm16556	3.57	0.0191			
Sycn	2.76	0.0191			
Slc15a1	3.24	0.0204			
Grin2b	3.81	0.022			
Cyp2d12	3.51	0.0223			
Gm11830	3.09	0.0236			
HOTTIP_2	7.46	0.0236			
HOXB13-AS1_1	6.11	0.0236			
Psg17	3.46	0.0236			
Cela1	2.1	0.0258			
Iqch	3.58	0.0258			
Foxq1	2.17	0.0259			
Ms4a10	1.48	0.0259			
Nt5c1a	3.4	0.026			
Il18	1.6	0.0264			
Myo16	2.71	0.0264			
Hoxd12	5.36	0.0271			
Brinp2	2.7	0.0291			
Ankdd1b	2.84	0.0292			
Defb45	4.97	0.0292			
Gp6	3.93	0.0328			
Ggh	2.36	0.0336			
Gm15401	1.69	0.0336			
A930011G23Rik	1.78	0.035			
Rdh16	1.75	0.035			
Hrg	4.05	0.0369			
1700042G15Rik	5.12	0.0411			
Wnt8b	3.96	0.0413			
Ctgf	1.44	0.0432			
Slc36a1	2.23	0.0432			
Nt5e	1.75	0.0434			
Sh3d21	1.87	0.0461			
Slc16a12	1.93	0.0466			
Gm16557	5.58	0.0469			
Ano4	2.8	0.0477			
Pla2g5	1.87	0.0477			
Gpr137b	2.36	0.0483			
Cd207	6.32	0.0493			
Gm17384	6.35	0.0494			
**Cluster 2**	**Up ↑**				
Gm10800	10	0.0023			
Gm10801	9.72	0.0034			
Gm21738	9.54	0.0035			
Gm26870	7.7	0.0191			
Gm10718	7.06	0.0236			
**Cluster 3**	**Up↑**				
Apon	2.18	0.0419			

One hundred thirty DEGs were selected by edgeR analysis of RNA sequence data (see Methods) and grouped into seven clusters by hierarchical clustering. Shown on the left are the three upregulated clusters (1–3), and shown on the right are the four dowregulated clusters (4–7).

NB regression (see Methods) selected four (1, 4, 6, 7) out of seven gene clusters (see Table 5) significantly (p-value < 0.05) in addition to APC genotype, which were positively associated with the relative abundance of Bacteroidetes. Among those four clusters, cluster 1 (coefficient 0.051) is composed of 100 out of 106 upregulated genes. The downregulated gene clusters 4,6,7 covers 22 out of 24 downregulated genes. While APC genotype had a dominant effect on the relative abundance of Bacteroidetes, detection of additional associations with mouse colonic gene expression, suggest that alterations in host colonic gene expression play a role in influencing mucosal associated microbial composition.

**Table 5:** Association between gene cluster expression (centroid medians), APC genotype and the relative abundance of Bacteroidetes in the proximal colonic mucosa of 6 week old APC^Min/+^ and WT mice.

Bacteroidetes	Coefficient	p-value
**APC genotype**	0.915	8.36E-21
**cluster1**	0.051	0.000632
**cluster4**	0.031	7.17E-10
**cluster6**	0.006	0.000556
**cluster7**	0.029	0.000158

The cluster medians of seven geneclusters along with APC genotype were used in the following model as described in *Methods*. The significant effects are **bolded**, with the threshold set as p-value <0.05. Regression coefficients are also reported as index of effect size.

Because elevated IL-1β levels have previously been reported in 18–25 week old APC^Min/+^ compared with WT mice, RT-PCR assays were conducted on proximal colonic RNA samples in all of the mice as previously described [15]. No significant difference was observed in the ΔCt_IL1β-actin_ values between APC^Min/+^ and WT mice (-13.5 vs. -16.2, p = 0.485). These values were both very low compared to that measured in DSS treated mice (-4.9), indicating that IL-1β was not highly expressed in the colons of either mouse group in our study.

## Discussion

Alterations in the gut microbiome (dysbiosis) have been reported in human colonic neoplasia and in mouse models [1–6, 47]. This study demonstrates that alterations in the gut microbiome, characterized by an increased relative abundance of *Bacteroidetes* spp. observed in association with intestinal neoplasias, actually precedes the development of microscopically detectable intestinal neoplasias in 6 week old APC^Min/+^ mice. Increased loads of *Bacteroidetes spp*. have been reported in another colitis-associated mouse model of colon cancer [47], and in some but not all studies of human colorectal neoplasia [1–6]. 16S rRNA sequence analysis revealed that the increased relative abundance of *Bacteroidetes spp*. corresponded primarily to an increased relative abundance of taxa within the uncultured family S24-7. Similar increases in S24-7 have also been reported in conventionally raised C57BL6 mice that were fed a high fat diet [48]. This association with intestinal dysbiosis is of interest, because increased dietary fat has previously been associated with increased number and /or size in both WT and APC^Min/+^ mice [49,50].

The relative abundance of the phylum *Tenericutes* observed in this study is higher than reported by some studies of C57Bl/6 mice [51], but similar to another study using C57Bl/6 mice purchased from the same vendor [52]. In this study, the APC^Min/+^ and WT mice were housed in separate cages, which could influence the reported microbial compositions [53, 54], possibly related to coprophagic behavior.

Alterations in gene expression have been previously reported in normal appearing mucosa of APC mutant mice after the development of intestinal polyposis [55]. We report differential expression of genes (DEG) in 6 week APC^Min/+^ mice prior to the detection of intestinal polyposis. In order to integrate host colonic gene expression with the microbial taxonomic data, we reduced the gene expression input variables by first selecting DEGs, reasoning that these genes would be most likely to be involved in disrupted colonic microbial interactions in the mutant mice. Variable dimensionality was further reduced by clustering the 130 DEGs into seven groups. The detection of significant associations between host colonic gene expression and the relative abundance of microbial taxa, after taking into consideration APC genotype, support the concept that host colonic microbial cross talk influences mucosal associated microbial composition. Cluster 4, which included downregulated genes encoding immunoglobulins and non-coding functional RNAs, demonstrated a significant linear relationship with the relative abundance of *Bacteroidetes* after controlling for APC genotype. The inverse correlation between immunoglobulin gene expression and the relative abundance of *Bacteroidetes spp*. is intriguing in light of previous reports linking an increased relative abundance of *Bacteroidetes spp*. with a reduction of immunoglobulin coated bacteria in humans [56, 57]. The observation that some of the non-coding RNAs in this cluster may be located in the mitochondria, is intriguing in light of the observation that mutated APC proteins in contrast to WT APC proteins are detected in mitochondria [58]

In summary, our results support the concept that APC haplo-insufficiency of the host colonic epithelial cell alters colonic microbial interactions prior to polyposis. It is thus conceivable that such microbiome changes contribute to the pathogenesis of colon cancer. An important corollary to such a notion would be that the colonic microbiome represents an important (and druggable) target for the prevention of colon cancer. Indeed, interventions directed at the microbiome (germ free and antibiotic treatment) have been reported to modulate tumor formation in mouse models of colon cancer [17]. However, it remains to be determined whether interventions directed at ameliorating dysbiosis in APC^Min/+^ mice, such as through probiotic, prebiotic or antibiotic interventions, could reduce tumor formation.
